# Supplementary material for: Targeting the pregnane X receptor using microbial metabolite mimicry
Source: EMBO Mol Med. 2020 Mar 10;12(4):e11621. doi: 10.15252/emmm.201911621 (PMC7136958; doi:10.15252/emmm.201911621)
Supplement: Supplementary file 2 — Expanded View Figures PDF [file EMMM-12-e11621-s002.pdf]

Expanded View Figures

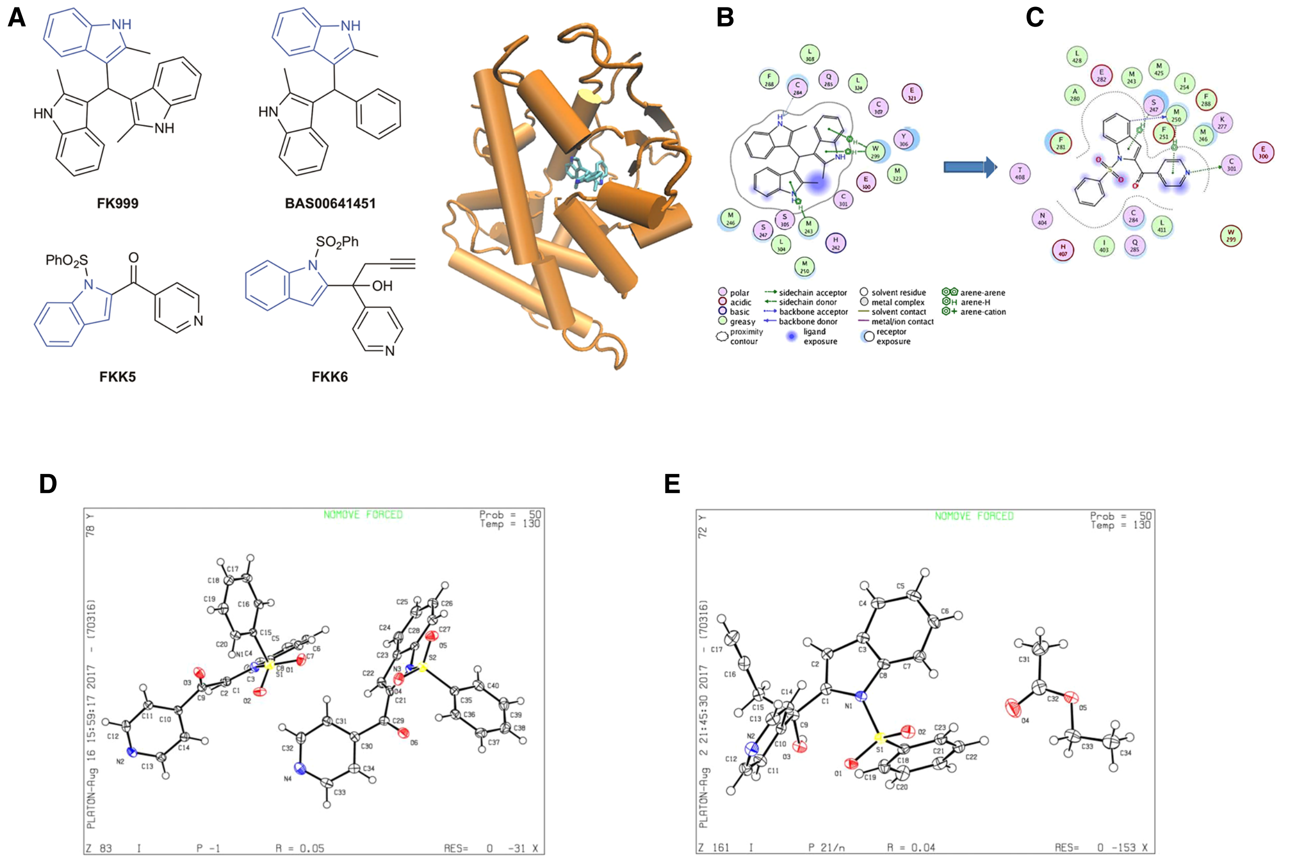

Figure EV1. FKK compound structures and their docked complex with hPXR.

A Chemical structures of FKK compounds and BAS00641451. Indole is colored blue. hPXR is shown as cartoon model and colored orange.

B, C FKK999 is shown as licorice sticks and colored atom type (C—cyan, N—blue, O—red). Hydrogen atoms are removed for clarity. Schematic representation of (B) FKK999 and (C) FKK5 in the binding pocket of hPXR (all residues within 4.5 Å (angstrom)). The legend below the figure describes the nature of interactions. (B, C) The figures were generated using the ligX module of MOE modeling package (ver 10.1).

D ORTEP drawing of FKK5. The crystal is non-merohedrally twinned, which causes the R_{int} value to be slightly higher than usual. There are two molecules in the asymmetric unit. They are related by inversion symmetry, but there does not appear to be a crystallographic inversion center between them; attempts at using a smaller unit cell or a higher symmetry space group were not successful.

E ORTEP drawing of FKK6. The compound crystallized in a centrosymmetric space group, indicating that the crystal contains a racemic mixture. There is one molecule of ethyl acetate co-crystallized with FKK6. The OH group forms an intermolecular hydrogen bond with the pyridine group.

Source data are available online for this figure.

 © 2020 The Authors

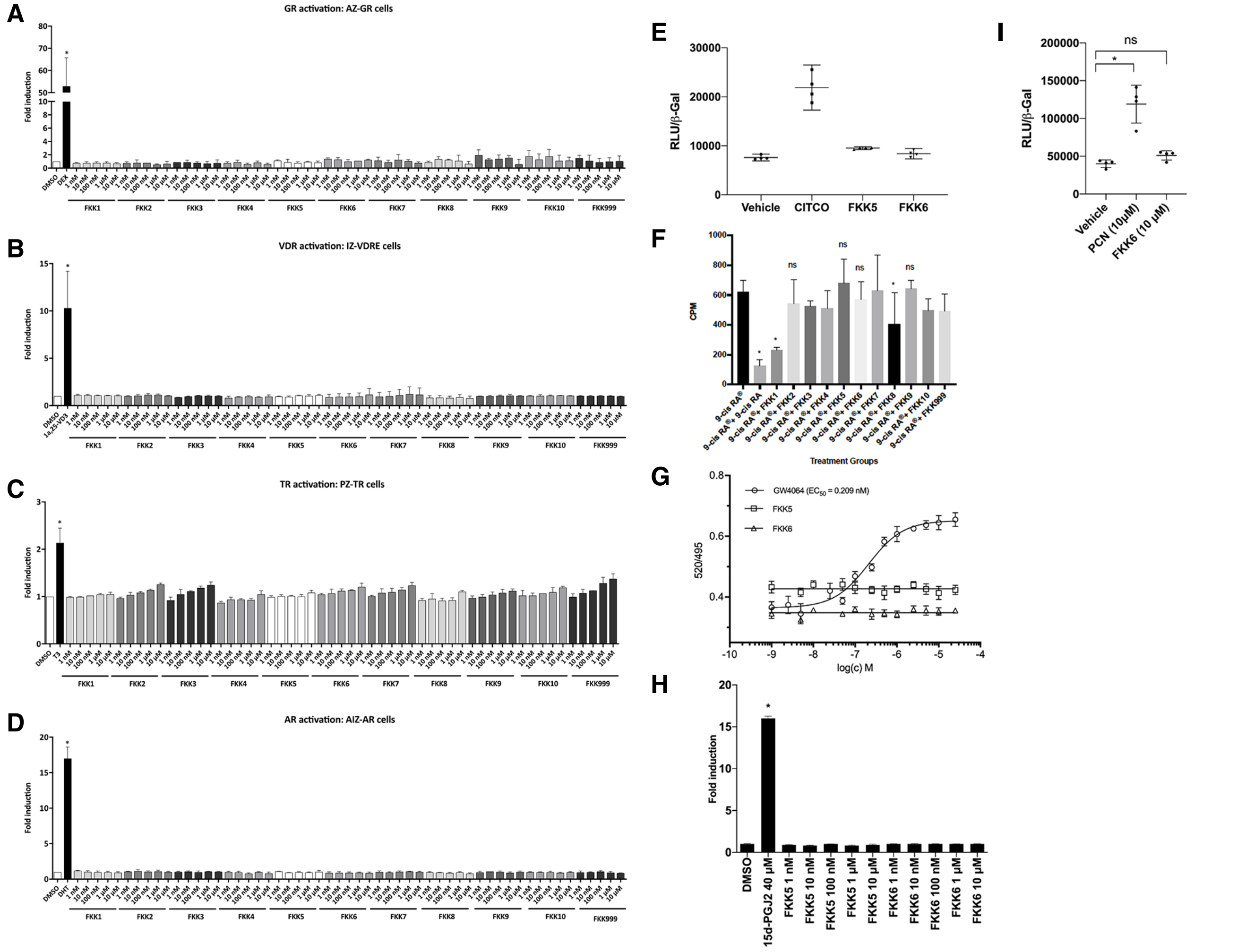

Figure EV2. FKK5 and FKK6 do not activate steroid and related nuclear receptors.

A–H Transcriptional activity of the respective nuclear receptors after incubations in the presence of their respective ligands. Transgenic AZ-GR, IZ-VDRE, PZ-TR, AIZ-AR, and PAZ-PPARγ cells were incubated with tested compounds and the model ligands, i.e., dexamethasone (DEX, 100 nM), calcitriol (1α,25-VD3, 50 nM), 3,5,3′-triiodothyronine (T3, 10 nM), dihydrotestosterone (DHT, 100 nM), and 15-deoxy-δ12,14-prostaglandin J2 (15d-PGJ2; 40 μM), respectively. (A) Glucocorticoid receptor; (B) vitamin D receptor; (C) thyroid receptor; (D) androgen receptor; and (H) peroxisome proliferator-activated receptor gamma (PPARγ). (A–D) The histogram depicts mean $\pm$ SD from experiments performed in two consecutive passages of cells. (H), Histogram depicts mean $\pm$ SD from one of two independent experiments each performed in triplicate. *$P < 0.05$, two-way ANOVA. (E) Human constitutive androstane receptor (hCAR) reporter activity in HEK293T cells. The RLU-to-beta-galactosidase ratio was calculated for CAR ligand CITCO, FKK5, FKK6, and vehicle control. These data represent one experiment performed in quadruplicate. Assays were performed at least two separate times, each in quadruplicate. (F) Displacement capacity of 9-*cis*-retinoic acid and FKK compounds tested for their potential capability to play a role as RXRs ligands. [11,12-^{3}H(N)] 9*c*RA (0.66 nM) was applied in all groups. Compounds from left: 9-*cis*-retinoic acid (39.94 nM), FKK1–FKK10 and FKK999 (39.94 nM) (**$P < 0.01$, *$P < 0.05$ vs. total [11,12-^{3}H(N)] 9*c*RA binding, Student's *t*-test). (G) FXR TR-FRET assay. TR/FRET ratio (520/495 nm) is plotted against concentration of compound(s). Half-maximal inhibitory concentrations IC_{50} were obtained from interpolated standard curves (sigmoidal, 4PL, variable slope); error bars show standard deviation of $n = 2$ independent experiments each with four technical replicates.

I Mouse pregnane X receptor (mPXR) reporter (MRP2) activity in HEK293T cells. The RLU-to-beta-galactosidase ratio was calculated for PXR ligand pregnane carbonitrile (PCN), FKK6, and vehicle control. These data represent one of two independent experiments, each performed in quadruplicate. The histogram depicts mean $\pm$ SD. *$P < 0.05$, one-way ANOVA.

Source data are available online for this figure.

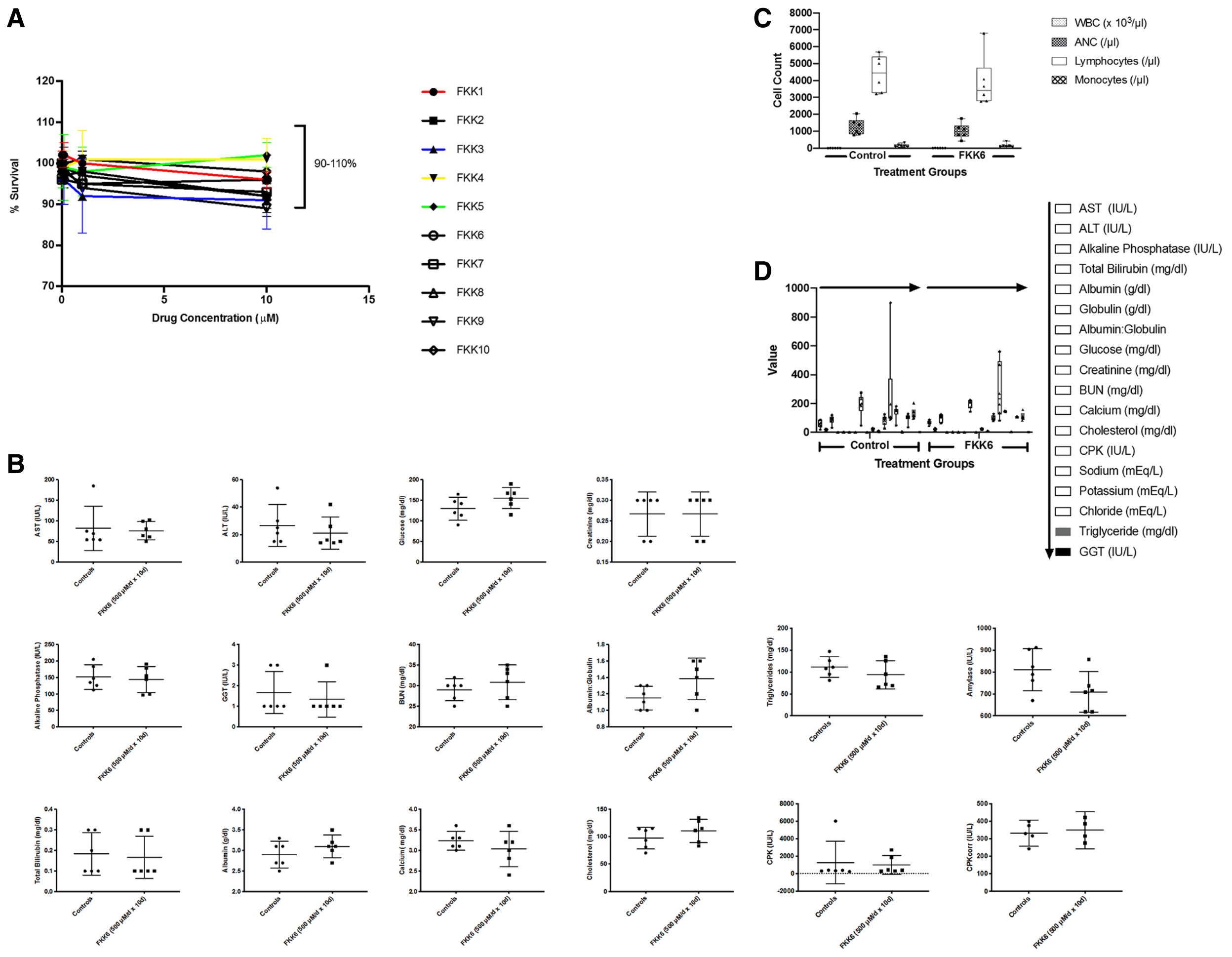

Figure EV3. FKK6 is not cytotoxic to cells and mice.

A Cell cytotoxicity (MTT) assays of FKK compounds in LS180 cells. Assays were repeated at least three independent times, each in quadruplicates. Data presented as mean (± SD).

B Serum chemistry panel of mice exposed to FKK6 (n = 6) or vehicle/controls (n = 6). Studies were performed on day 10.

C, D Complete blood count (C) and serum (D) Chemistry panel of mice exposed to FKK6 (n = 6) or vehicle /controls (n = 6). Studies were performed on day 30.

Data information: (B–D) The data are plotted as mean (95% CI); not significant ($P > 0.05$) in any panel, Student's t-test. CPKcorr, corrected CPK values.

Source data are available online for this figure.

 © 2020 The Authors

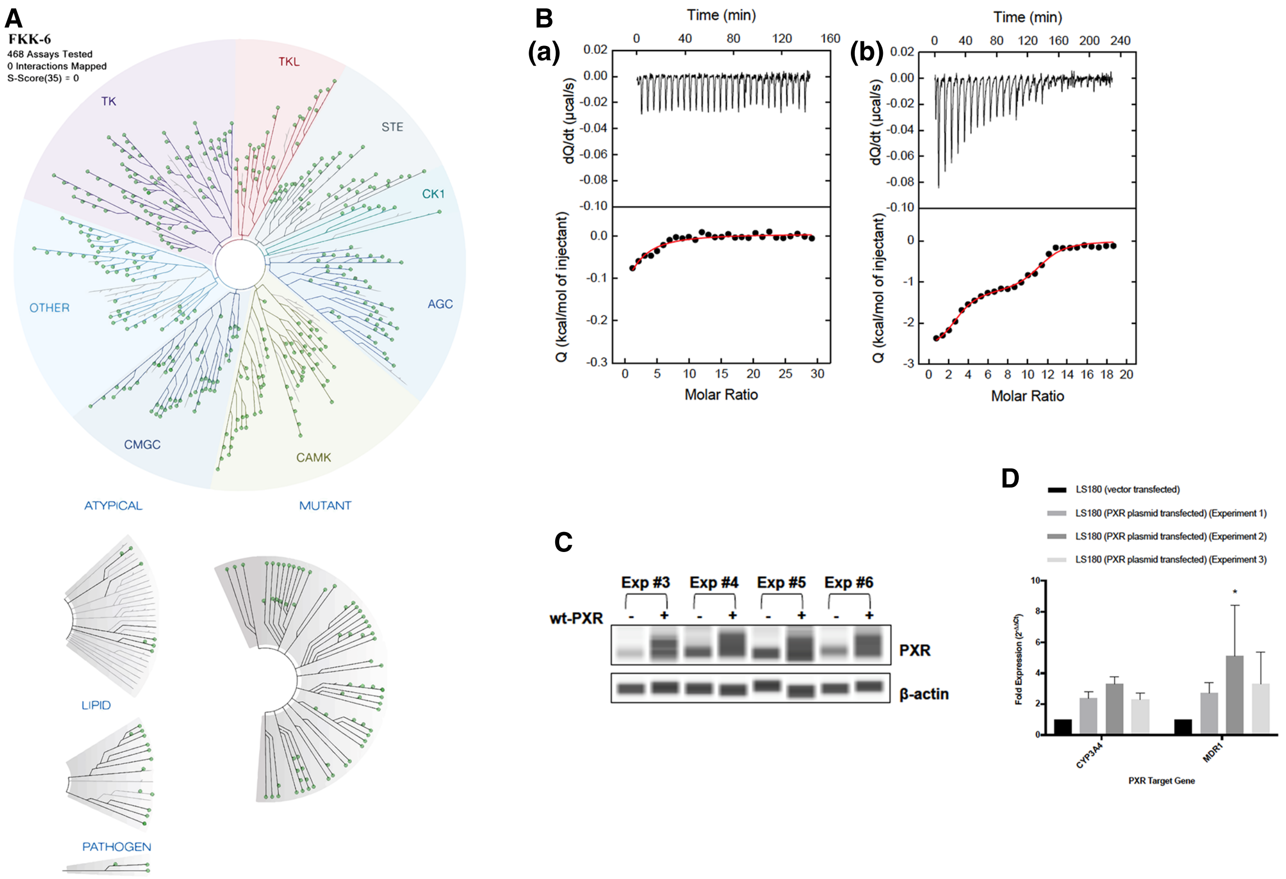

Figure EV4. FKK6 does not inhibit a broad array of kinase enzymes.

A TREEspotTM interaction maps. The distribution of kinases and its activity in the presence of FKK6 (10 μM) in the screening panel is represented by green dots (kinase activity percent control > 35%) on a dendrogram representing the human kinome (kinome image is reproduced courtesy of TREE*spot*™, DiscoverX proprietary data visualization tool, KINOME*scan*).

B Microcalorimetric titrations of PXR ligand-binding domain (LBD) with (a) 3-indole propionic acid and (b) rifampicin.

C Confirmation of transfection PXR in LS180 cell line. The levels of PXR and β-actin proteins were determined by quantitative automated Western blot analyzer Sally Sue from the ProteinSimple software. wt-PXR, LS180 cells transiently transfected with wild-type human PXR plasmid; Exp (#number), number of the experiment.

D PXR expression in LS180 cells induces CYP3A4 and MDR1 mRNA expression. Expression data were normalized to the housekeeping gene GAPDH. Data are expressed as fold induction ± SD over the mock-transfected cells. Differences were tested using one-way ANOVA with Dunnett's *post hoc* test, $^*P < 0.01$.

Source data are available online for this figure.

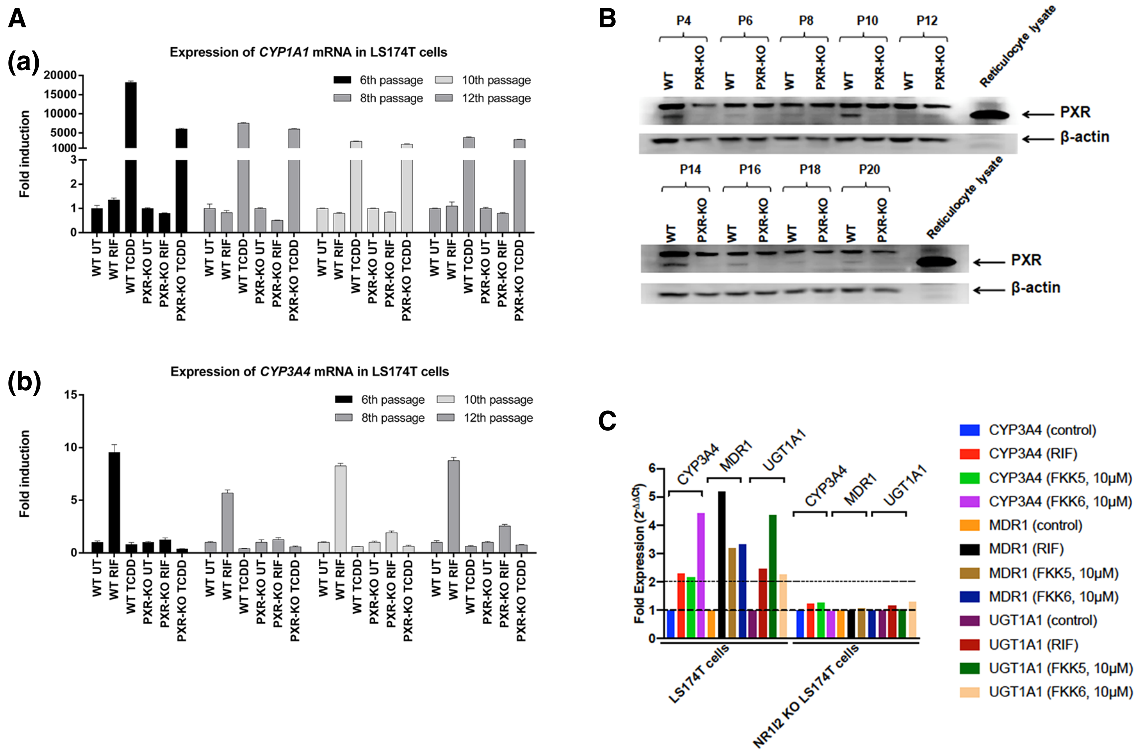

Figure EV5. LS174T PXR-knockout (KO) cells are not responsive to PXR ligands.

A Expression of CYP1A1 and CYP3A4 mRNA in PXR-knockout and wild-type LS174T. Cells were treated with vehicle (UT; DMSO; 0.1% v/v), rifampicin (RIF; 10 µM), or 2,3,7,8-tetrachlordibenzodioxin (TCDD; 5 nM) for 24 h. Experiment was performed in 4 independent cell passages (6th, 8th, 10th, and 12th). The mRNA expression of CYP1A1 and CYP3A4 mRNA was determined by RT–PCR, and data were normalized to GAPDH mRNA level. (a) CYP1A1 mRNA. (b) CYP3A4 mRNA. Data are expressed as mean ± SD.

B Immunoblot for PXR protein expression in PXR wild-type and knockout (KO) cells. Experiment was performed in nine independent cell passages (P).

C Fold mRNA expression of PXR target genes. Each tissue PCR was repeated in quadruplicate. The expression was normalized to internal control, GAPDH. The entire experiment was repeated $n > 2$ for reproducibility. The data shown are one representative experiment.

Source data are available online for this figure.

 © 2020 The Authors
