# Supplementary material for: Targeting the pregnane X receptor using microbial metabolite mimicry
Source: EMBO Mol Med. 2020 Mar 10;12(4):e11621. doi: 10.15252/emmm.201911621 (PMC7136958; doi:10.15252/emmm.201911621)
Supplement: Supplementary file 3 — Table EV1 [file EMMM-12-e11621-s003.docx]

**Table EV1.** Crystal, intensity collection, and refinement data.

|  | **FKK5** |
| --- | --- |
| lattice | Triclinic |
| formula | C_20_H_14_N_2_O_3_S |
| formula weight | 362.39 |
| space group | *P-1* |
| *a*/Å | 9.4678(9) |
| *b*/Å | 10.7465(10) |
| *c*/Å | 17.3877(19) |
| α/˚ | 88.938(7) |
| β/˚ | 80.003(7) |
| γ/˚ | 71.208(6) |
| *V*/Å^3^ | 1648.1(3) |
| *Z* | 4 |
| temperature (K) | 130(2) |
| radiation (λ, Å) | 0.71073 |
| ρ (calcd.) g cm^-3^ | 1.461 |
| μ (Mo Kα), mm^-1^ | 0.220 |
| θ max, deg. | 25.164 |
| no. of data collected | 137579 |
| no. of data | 5868 |
| no. of parameters | 470 |
| *R_1_* [*I > 2σ(I)*] | 0.0512 |
| *wR_2_* [*I > 2σ(I)*] | 0.1268 |
| *R_1_* [all data] | 0.0658 |
| *wR_2_* [all data] | 0.1333 |
| GOF | 1.082 |
| *R_int_* | 0.1420 |
