# Supplementary material for: Targeting the pregnane X receptor using microbial metabolite mimicry
Source: EMBO Mol Med. 2020 Mar 10;12(4):e11621. doi: 10.15252/emmm.201911621 (PMC7136958; doi:10.15252/emmm.201911621)
Supplement: Supplementary file 4 — Table EV2 [file EMMM-12-e11621-s004.docx]

|  | **FKK6•EtOAc** |
| --- | --- |
| lattice | Monoclinic |
| formula | C_27_H_26_N_2_O_5_S |
| formula weight | 490.56 |
| space group | *P2_1_/n* |
| *a*/Å | 7.7834(13) |
| *b*/Å | 19.087(3) |
| *c*/Å | 16.355(3) |
| α/˚ | 90 |
| β/˚ | 100.528(4) |
| γ/˚ | 90 |
| *V*/Å^3^ | 2388.8(7) |
| *Z* | 4 |
| temperature (K) | 130(2) |
| radiation (λ, Å) | 0.71073 |
| ρ (calcd.) g cm^-3^ | 1.364 |
| μ (Mo Kα), mm^-1^ | 0.178 |
| θ max, deg. | 27.601 |
| no. of data collected | 39284 |
| no. of data | 5140 |
| no. of parameters | 326 |
| *R_1_* [*I > 2σ(I)*] | 0.0426 |
| *wR_2_* [*I > 2σ(I)*] | 0.0816 |
| *R_1_* [all data] | 0.0814 |
| *wR_2_* [all data] | 0.0942 |
| GOF | 1.002 |
| *R_int_* | 0.0722 |

**Table EV2.** Crystal, intensity collection, and refinement data.
