# Supplementary material for: Targeting the pregnane X receptor using microbial metabolite mimicry
Source: EMBO Mol Med. 2020 Mar 10;12(4):e11621. doi: 10.15252/emmm.201911621 (PMC7136958; doi:10.15252/emmm.201911621)
Supplement: Supplementary file 5 — Table EV3 [file EMMM-12-e11621-s005.docx]

**Table EV3.** 10 Day toxicity study in mice

| **Organ** | **Vehicle**  **n=6*** | **FKK6**  **n=6** |
| --- | --- | --- |
| Heart | Minimal lymphoplasmacytic infiltrates  (n=1) | – |
| Coronary Arteries/Aortic Root | – ** | – |
| Liver | Mild periportal inflammation  (n=5),  Hepatocyte necrosis  (n=3) | Mild periportal inflammation  (n=5) |
| Kidney | Degenerative tubules/vacuoles  (rare)  (n=6) | Degenerative tubules/vacuoles  (rare)  (n=6) |
| Lung | Mild leukocytes  (n=3) | Mild leukocytes  (n=2) |
| Stomach | – | – |
| Small Intestine | – | – |
| Large Intestine | – | – |
| Cecum | – | – |
| Colon | – | – |
| Spleen | Mild lymphoid hyperplasia  (n=2) | Mild lymphoid hyperplasia  (n=2) |
| Bone Marrow | – | – |

*One mouse was found deceased during day 10 cage inspection. All other mice examined were in good body condition with no minimal autolysis and no gross lesions.

**Aortic root not visualized in (n=1) mouse.
