# Supplementary material for: Targeting the pregnane X receptor using microbial metabolite mimicry
Source: EMBO Mol Med. 2020 Mar 10;12(4):e11621. doi: 10.15252/emmm.201911621 (PMC7136958; doi:10.15252/emmm.201911621)
Supplement: Supplementary file 6 — Table EV4 [file EMMM-12-e11621-s006.docx]

**Table EV4.** 30 Day toxicity study in mice

| **Organ** | **Vehicle**  **n=6** | **FKK6**  **n=6** |
| --- | --- | --- |
| Heart | – | – |
| Coronary Arteries/Aortic Root | – | – |
| Liver | Mild perivascular lymphohistiolytic aggregates  (n=3) | Mild perivascular lymphohistiocytic aggregates  (n=4) |
| Kidney | Degenerative tubules/vacuoles  (rare)  (n=1) | – |
| Lung | Mild leukocytes  (n=3) | Mild leukocytes  (n=2) |
| Stomach | Focal neutrophilic aggregate  (n=1) | – |
| Small Intestine | – | – |
| Large Intestine | – | – |
| Cecum | Moderate lymphoid hyperplasia  (n=1) | – |
| Colon | – | – |
| Spleen | Mild lymphoid hyperplasia  (n=2) | Mild lymphoid hyperplasia  (n=2) |
| Bone Marrow | – | – |
