# Supplementary material for: Targeting the pregnane X receptor using microbial metabolite mimicry
Source: EMBO Mol Med. 2020 Mar 10;12(4):e11621. doi: 10.15252/emmm.201911621 (PMC7136958; doi:10.15252/emmm.201911621)
Supplement: Supplementary file 7 — Table EV5 [file EMMM-12-e11621-s007.docx]

**Table EV5.** Kinase Screen

**Table EV5A.** S-Score (468 Kinase Screen) Results for FKK6

| **Compound Name** | **Selectivity Score Type** | **Number of Hits** | **Number of Non-mutant Kinases** | **Screening Concentration**  **(μM)** | **Selectivity Score (S-Score)** |
| --- | --- | --- | --- | --- | --- |
| FKK6 | S(35) | 0 | 403 | 10 | 0 |
| FKK6 | S(10) | 0 | 403 | 10 | 0 |
| FKK6 | S(1) | 0 | 403 | 10 | 0 |

**Table EV5B.** Select Kinase Screen Results for FKK6

| **Acetyltransferase** | **Substrate** | **FKK6(data1, Exp 1)** | **FKK6(data2, Exp 1)** | **FKK6(data1, Exp 2)** | **FKK6(data2, Exp 2)** |
| --- | --- | --- | --- | --- | --- |
| P300 | Histone H3 | 100.32 | 97.73 | 98.72 | 95.38 |
| pCAF | Histone H3 | 100.32 | 101.68 | 104.61 | 104.47 |
|  |  |  |  |  |  |
|  | % Enzyme Activity to DMSO controls | | |  |  |
|  | Positive controls used (IC50) C646, Anacardic acid (full data available upon request) | | | | |
|  | | | | | |
| **Enzyme** | **FKK6(data1, Exp 1)** | **FKK6(data2, Exp 1)** | **FKK6(data1, Exp 2)** | **FKK6(data2, Exp 2)** |  |
| PARP 1 | 99.18 | 98.25 | 91.38 | 91.03 |  |
|  |  |  |  |  |  |
|  | % Enzyme Activity to DMSO controls | | | |  |
|  | Positive controls used (IC50) PJ34, PJ35 (full data available upon request) | | | | |
|  | | | | | |
| **Methyltransferase** | **substrate** | **FKK6(data1, Exp 1)** | **FKK6(data2, Exp 1)** | **FKK6(data1, Exp 2)** | **FKK6(data2, Exp 2)** |
| PRMT1 | Histone H4 | 86.43 | 88.77 | 78.47 | 81.3 |
|  |  |  |  |  |  |
|  | % Enzyme Activity to DMSO controls | | | |  |
|  | Positive control is SAH, (S-(5’-Adenosyl)-L-homocysteine) (full data available upon request) | | | | |
|  | | | | | |
| **Histone Deacetylases** | **substrate** | **FKK6(data1, Exp 1)** | **FKK6(data2, Exp 1)** | **FKK6(data1, Exp 2)** | **FKK6(data2, Exp 2)** |
| HDAC1 | Peptide (Ac) | 106.36 | 101.46 | 89.76 | 86.7 |
| HDAC2 | Peptide (Ac) | 86.27 | 78.19 | 83.24 | 88.71 |
| HDAC3 | Peptide (Ac) | 122.38 | 116.9 | 114.05 | 107.82 |
|  |  |  |  |  |  |
|  | % Enzyme Activity to DMSO controls | | | |  |
|  | Positive controls used (IC50) Trichostatin A (full data available upon request) | | | | |
|  |  |  |  |  |  |
| **Enzyme** | **Substrate** | **FKK6(data1, Exp 1)** | **FKK6(data2, Exp 1)** | **FKK6(data1, Exp 2)** | **FKK6(data2, Exp 2)** |
| SIRT1 | Acetylated peptide | 100.18 | 100.03 | 99.84 | 94.47 |
|  |  |  |  |  |  |
|  | % Enzyme Activity to DMSO controls | | | |  |
|  | Positive controls used (IC50) Suramin (full data available upon request) | | | | |

**Table EV5C.** Select Kinase Screen Results for FKK5

**Top**

| ALB002-01-p-00001 Study Results | | | | |
| --- | --- | --- | --- | --- |
| Table 1 – Matrix of Compound Screen for ALB002-01-p-00001 | | | | |
| Target | FKK5 | |  |  |
| Gene Symbol | %Ctrl @ 10000 nM | %Ctrl @ 25000 nM |  |  |
| CDK2 | 100 | 89 |  |  |
| CDK4 | 100 | 95 |  |  |
| CDK5 | 77 | 61 |  |  |
| ERK1 | 86 | 46 |  |  |
| ERK2 | 99 | 94 |  |  |
| ERK3 | 100 | 80 |  |  |
| ERK4 | 97 | 89 |  |  |
| ERK5 | 94 | 100 |  |  |
| ERK8 | 97 | 75 |  |  |
| GSK3A | 79 | 49 |  |  |
| GSK3B | 100 | 98 |  |  |
| IKK-alpha | 96 | 83 |  |  |
| JNK1 | 91 | 66 |  |  |
| JNK2 | 95 | 74 |  |  |
| PKAC-alpha | 100 | 85 |  |  |
| PKAC-beta | 100 | 92 |  |  |
| S6K1 | 79 | 71 |  |  |
|  | | | | |
| % Ctrl Legend |  |  |  |  |
| 0 ≤ x ≤ .1 | .1 ≤ x < 1 | 1 ≤ x < 10 | 10 ≤ x < 35 | X ≥35 |

**Bottom**

| Target | FKK5 | |  |  |
| --- | --- | --- | --- | --- |
| Gene Symbol | %Ctrl @ 10000 nM | %Ctrl @ 25000 nM |  |  |
| CDK2 | 74 | 63 |  |  |
| CDK4 | 98 | 90 |  |  |
| CDK5 | 100 | 100 |  |  |
| ERK1 | 100 | 100 |  |  |
| ERK2 | 100 | 85 |  |  |
| ERK3 | 88 | 93 |  |  |
| ERK4 | 82 | 91 |  |  |
| ERK5 | 79 | 92 |  |  |
| ERK8 | 71 | 65 |  |  |
| GSK3A | 92 | 77 |  |  |
| GSK3B | 92 | 90 |  |  |
| IKK-alpha | 86 | 100 |  |  |
| JNK1 | 95 | 85 |  |  |
| JNK2 | 88 | 80 |  |  |
| PKAC-alpha | 100 | 95 |  |  |
| PKAC-beta | 57 | 48 |  |  |
| S6K1 | 100 | 89 |  |  |
|  | | | | |
| % Ctrl Legend |  |  |  |  |
| 0 ≤ x ≤ .1 | .1 ≤ x < 1 | 1 ≤ x < 10 | 10 ≤ x < 35 | X ≥35 |
