# Supplementary figures and images for: Targeting the pregnane X receptor using microbial metabolite mimicry
Source: EMBO Mol Med. 2020 Mar 10;12(4):e11621. doi: 10.15252/emmm.201911621 (PMC7136958; doi:10.15252/emmm.201911621)

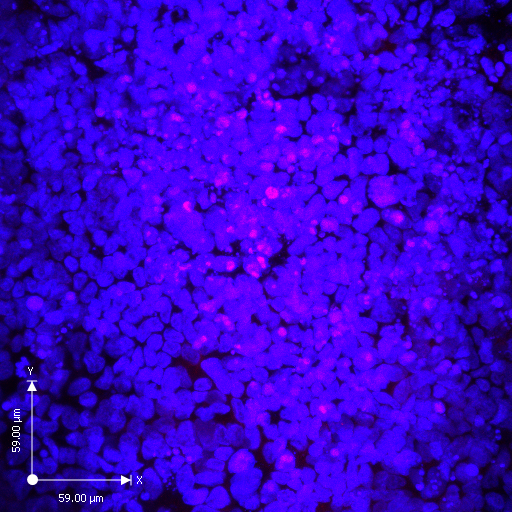

Supplement: Supplementary file 12 — Source Data for Figure 3 [file EMMM-12-e11621-s011.zip › EMM-2019-11621-T_Fig_3_raw_images/EMM-2019-11621-T_12_hr_Caco-2_CK.tif]

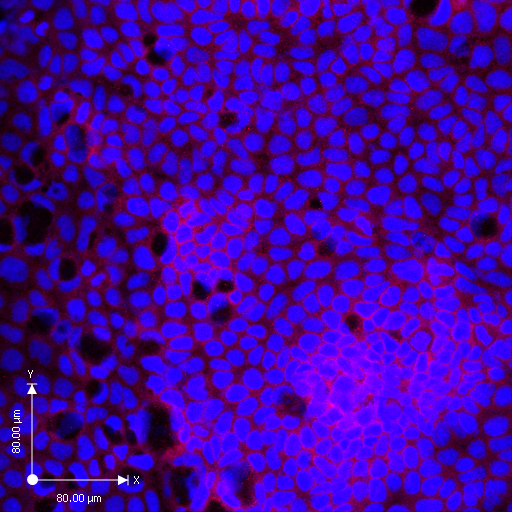

Supplement: Supplementary file 12 — Source Data for Figure 3 [file EMMM-12-e11621-s011.zip › EMM-2019-11621-T_Fig_3_raw_images/EMM-2019-11621-T_12_hr_Caco-2_CK_FKK6_25.tif]

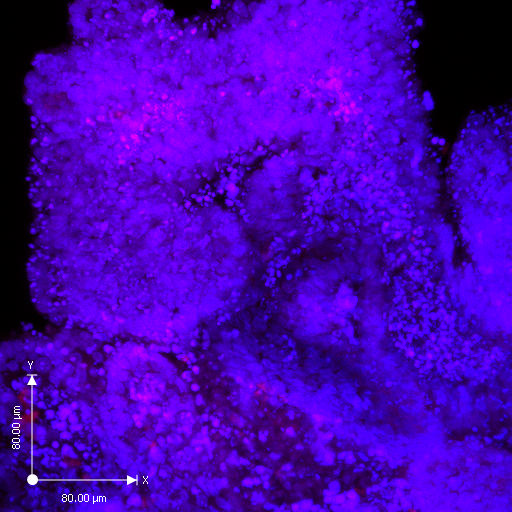

Supplement: Supplementary file 12 — Source Data for Figure 3 [file EMMM-12-e11621-s011.zip › EMM-2019-11621-T_Fig_3_raw_images/EMM-2019-11621-T_12_hr_HIO_CK.tif]

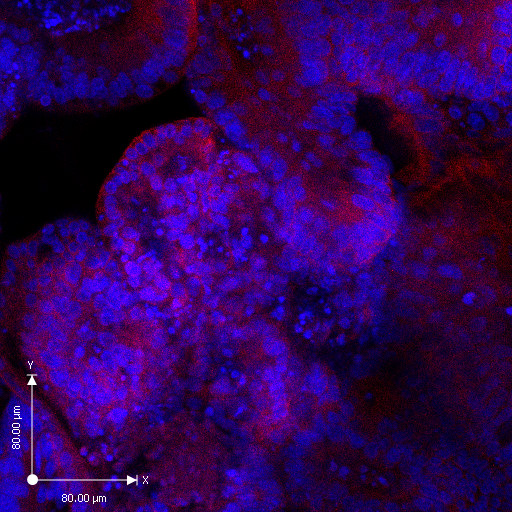

Supplement: Supplementary file 12 — Source Data for Figure 3 [file EMMM-12-e11621-s011.zip › EMM-2019-11621-T_Fig_3_raw_images/EMM-2019-11621-T_12_hr_HIO_CK_FKK5_25.tif]

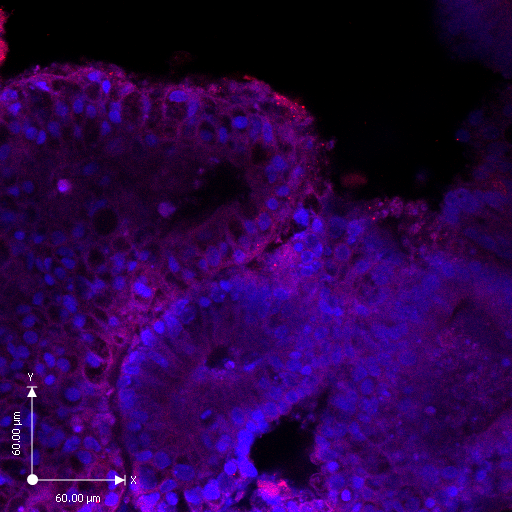

Supplement: Supplementary file 12 — Source Data for Figure 3 [file EMMM-12-e11621-s011.zip › EMM-2019-11621-T_Fig_3_raw_images/EMM-2019-11621-T_12_hr_HIO_CK_FKK6_25.tif]

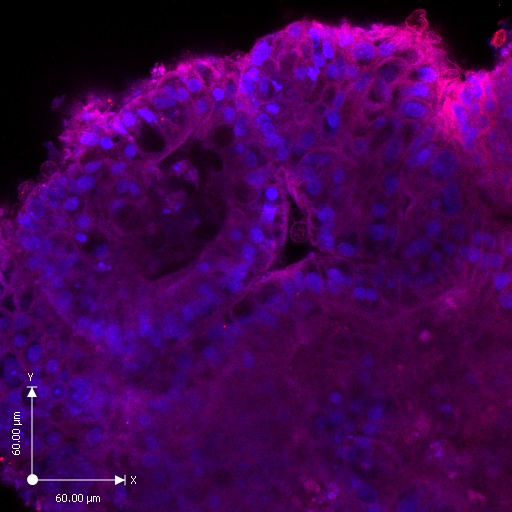

Supplement: Supplementary file 12 — Source Data for Figure 3 [file EMMM-12-e11621-s011.zip › EMM-2019-11621-T_Fig_3_raw_images/EMM-2019-11621-T_12_hr_HIO_no_CK.tif]

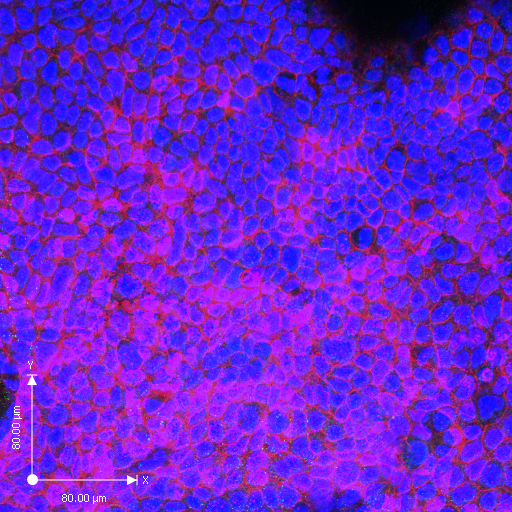

Supplement: Supplementary file 12 — Source Data for Figure 3 [file EMMM-12-e11621-s011.zip › EMM-2019-11621-T_Fig_3_raw_images/EMM-2019-11621-T_2_hr_Caco-2_CK.tif]

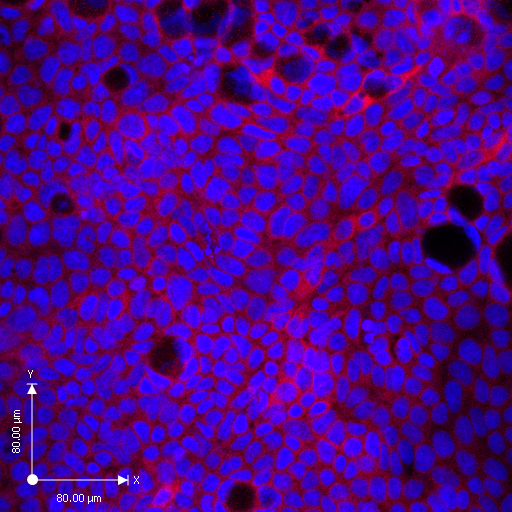

Supplement: Supplementary file 12 — Source Data for Figure 3 [file EMMM-12-e11621-s011.zip › EMM-2019-11621-T_Fig_3_raw_images/EMM-2019-11621-T_2_hr_Caco-2_CK_FKK5_25.tif]

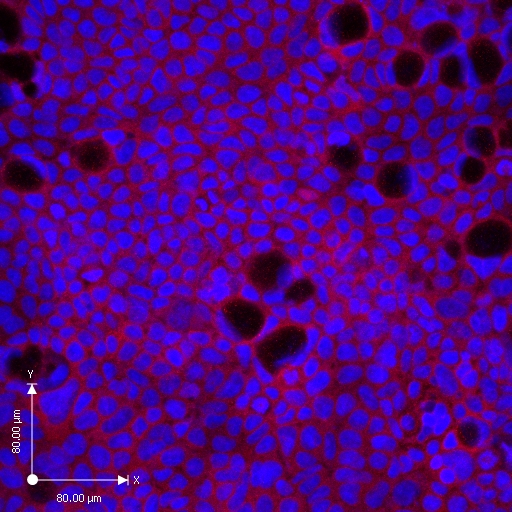

Supplement: Supplementary file 12 — Source Data for Figure 3 [file EMMM-12-e11621-s011.zip › EMM-2019-11621-T_Fig_3_raw_images/EMM-2019-11621-T_2_hr_Caco-2_CK_FKK6_25.tif]

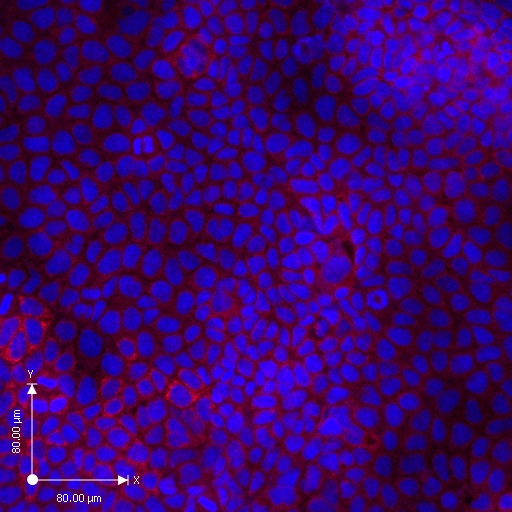

Supplement: Supplementary file 12 — Source Data for Figure 3 [file EMMM-12-e11621-s011.zip › EMM-2019-11621-T_Fig_3_raw_images/EMM-2019-11621-T_2_hr_Caco-2_no_CK.tif]

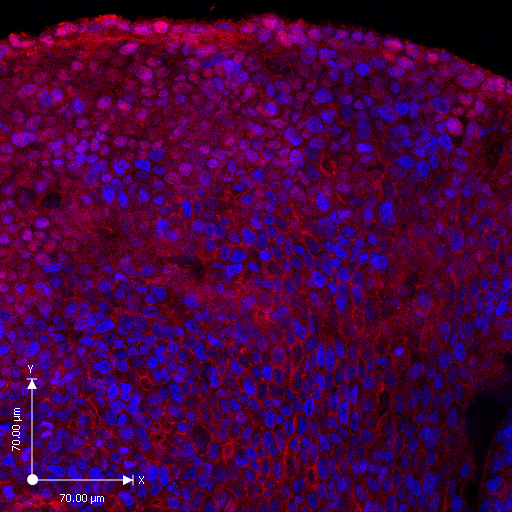

Supplement: Supplementary file 12 — Source Data for Figure 3 [file EMMM-12-e11621-s011.zip › EMM-2019-11621-T_Fig_3_raw_images/EMM-2019-11621-T_2_hr_HIO_CK.tif]

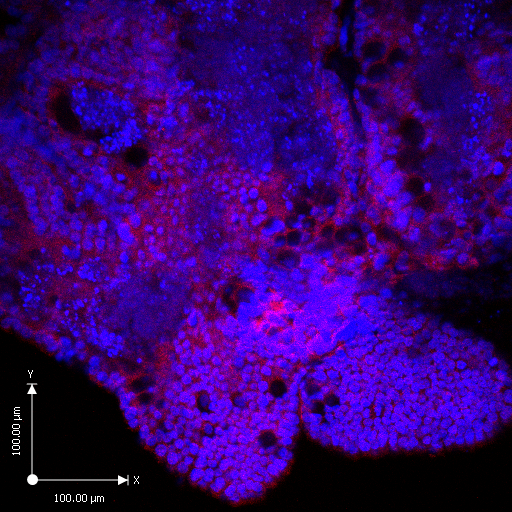

Supplement: Supplementary file 12 — Source Data for Figure 3 [file EMMM-12-e11621-s011.zip › EMM-2019-11621-T_Fig_3_raw_images/EMM-2019-11621-T_2_hr_HIO_CK_FKK5_25.tif]

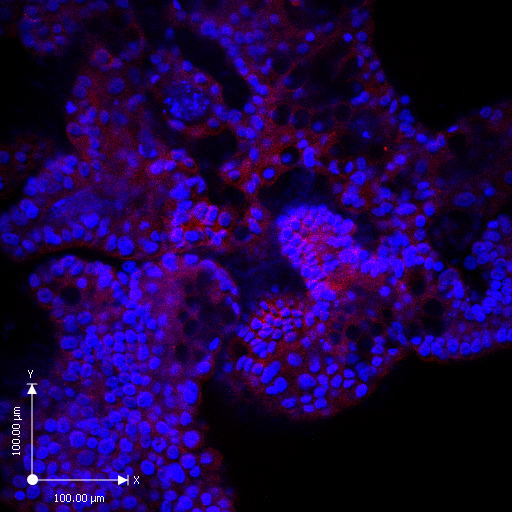

Supplement: Supplementary file 12 — Source Data for Figure 3 [file EMMM-12-e11621-s011.zip › EMM-2019-11621-T_Fig_3_raw_images/EMM-2019-11621-T_2_hr_HIO_CK_FKK6_25.tif]

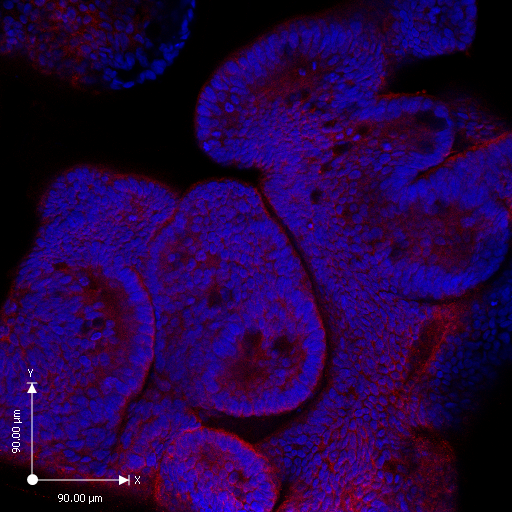

Supplement: Supplementary file 12 — Source Data for Figure 3 [file EMMM-12-e11621-s011.zip › EMM-2019-11621-T_Fig_3_raw_images/EMM-2019-11621-T_2_hr_HIO_no_CK.tif]
